# Extract of a Letter from Mr. Thomas Christie, Surgeon of the 80th Regiment, to Dr. Saunders, New Broad Street, London; Dated Trincomale, Island of Ceylon, May 21st, 1798

**Published:** 1799-08

**Authors:** 


FOR THE MEDICAL AND PHYSICAL JOURNAL.

*Extract of a Letter from Mr.* THOMAS CHRISTIE, *Surgeon of the 80th Regiment, to Dr.* SAUNDERS, *New Broad Street, London; dated Trincomale, Iſland of Ceylon, May 21ſt,* 1798.

"ON our firſt arrival at this ſtation, which is accounted one of the moſt unhealthy in India, we were very ſickly: of late, however, we are become extremely healthy, have not many ſick, and but few caſualties.—During my reſidence, although ſhort, in India, I have had conſiderable experience in the endemic diſeaſes of the country, particularly in hepatitis, and have had frequent opportunities of obſerving, in my own practice, the great juſtice and accuracy of your valuable remarks on that complaint.

"As I had for ſome time the care of the whole garriſon here, I had then an excellent opportunity of obſerving the comparative frequency of the diſeaſe, and violence of the ſymptoms, among the men lately arrived from Europe, the Europeans long in India, and the native troops.

"I

"I found, that among the men of the 80th regiment, for the firſt ſix or eight months, the diſeaſe was much more frequent, much more violent in its ſymptoms, ſhewed more tendency to ſuppuration, and was more ſudden in its criſis, than with the Company's European troops, who had been long in India, although the latter were the moſt debauched. Amongſt the natives, hepatitis does not ſo often occur; out of the thouſand native troops, I did not, in the courſe of three months, meet with more than two caſes of liver complaints, which is comparatively a very ſmall proportion.

"The following inſtance is ſtrongly a proof of your propoſition (part 5. ſect. 1. chap. 5.), with reſpect to the propenſity of the inflammation to the ſtomach, cauſing a conſtant reaching; it alſo ſeems to ſhew, that all the ſuppoſed pathognomic ſymptoms are not preſent in every inſtance of hepatitis: Corporal Potter, of the 80th regiment, a healthy young man, was attacked about the 6th of November, 1797, with ſymptoms of pyrexia, attended with pain at the pit of the ſtomach, dyſpnœa, and almoſt conſtant vomiting. As he had no cough, or affection of the bowels, he was treated as for an affection of the liver, although no tumour or particular pain was obſervable upon preſſure of the right hypochondrium, nor did he complain of the pain extending to the ſhoulder till within three days of his death, which happened on the 20th of November.

"Upon opening the abdomen after death, and raiſing the ſternum, I found the liver of its natural ſize, and in its uſual ſituation, without any adheſions between its convex ſurface and the abdominal perinæum, ſo that I began to conceive I had been miſtaken in my opinion of the caſe, till obſerving the ſtomach particularly prominent, and ſome adheſion between it and the concave ſurface of the liver, I ſeparated theſe with my fingers, when I found nearly a quart of well-formed pus contained between the ſtomach and the concave ſurface of the liver, a part of which latter was corroded, but the reſt of that organ, as well as the ſtomach and other viſcera, were in a ſound ſtate.

"I have made a point of opening every perſon who has died of the liver-complaint, while under my care; and amongſt the men of the 80th regiment, who were lately arrived from Europe, I did not find one out of twelve inſtances in which ſuppuration had not exiſted in ſome part or other of the liver. Suppuration, I have every reaſon to believe, is not near ſo frequent amongſt the natives, or Europeans who have been long in the country; and, indeed, amongſt the men of the 80th regiment, who have now been above fifteen months in India, I find that already the diſeaſe puts on a different form, becomes leſs frequent, more ſlow in its progreſs, and ſhews much leſs tendency tendency to run into fuppuration. On my firft coming here, I had originally fixteen or feventeen men in the hofpital with hepatitis—I have now feldom more than fix or feven, and have not loft a man from the complaint for the laft two months, although we are now ftronger in men than we were at that time, having been reinforced with drafts from fome old regiments. From the mode of recruiting the army here, it feldom happens that the care of fo many Europeans (about 800), juft arrived in India, falls to the charge of one perfon, at one time; I therefore thought that thefe few remarks, as they relate to Europeans lately arrived in India, might be acceptable to you.

"There are many marfhes, and much brufh-wood in the vicinity of the fort; the atmofphere is moift; and moft of the difeafes here are thofe of debility—to which I find the private men, as living worfe, are much more fubject than the officers. The fever-complaint has, however, I think, attacked a greater proportion of officers than men.

"I ought to obferve, that the fever-complaint is a familiar phrafe in India for hepatitis; from inadvertency of making ufe too often of that indefinite term; but I always mean hepatitis, both of the acute and chronic kind.

"In agues, which are very frequent here, I have had an opportunity of making a comparative trial of the pale red, and yellow bark, and from my own experience, have not the leaft hefitation of giving the preference to the laft."

